# Non-Coding RNAs in Saliva: Emerging Biomarkers for Molecular Diagnostics

**DOI:** 10.3390/ijms16048676

**Published:** 2015-04-17

**Authors:** Blanca Majem, Marina Rigau, Jaume Reventós, David T. Wong

**Affiliations:** 1 Research Unit in Biomedicine and Translational Oncology, Lab 209, Collserola Building, Vall Hebron Research Institute (VHIR) and University Hospital, Pg. Vall Hebron 119-129, 08035 Barcelona, Spain; E-Mails: blanca.majem@vhir.org (B.M.); marina.rigau@vhir.org (M.R.); jaume.reventos@vhir.org (J.R.); 2 IDIBELL-Bellvitge Biomedical Research Institute & Universitat Internacional de Catalunya, 08908 Barcelona, Spain; 3 Center for Oral/Head & Neck Oncology Research, University of California, Los Angeles, CA 90095, USA

**Keywords:** saliva, liquid biopsy, body fluid, disease, diagnostics, non-invasiveness, biomarkers, non-coding RNA (ncRNAs), small ncRNAs, long ncRNAs

## Abstract

Saliva is a complex body fluid that comprises secretions from the major and minor salivary glands, which are extensively supplied by blood. Therefore, molecules such as proteins, DNA, RNA, *etc.*, present in plasma could be also present in saliva. Many studies have reported that saliva body fluid can be useful for discriminating several oral diseases, but also systemic diseases including cancer. Most of these studies revealed messenger RNA (mRNA) and proteomic biomarker signatures rather than specific non-coding RNA (ncRNA) profiles. NcRNAs are emerging as new regulators of diverse biological functions, playing an important role in oncogenesis and tumor progression. Indeed, the small size of these molecules makes them very stable in different body fluids and not as susceptible as mRNAs to degradation by ribonucleases (RNases). Therefore, the development of a non-invasive salivary test, based on ncRNAs profiles, could have a significant applicability to clinical practice, not only by reducing the cost of the health system, but also by benefitting the patient. Here, we summarize the current status and clinical implications of the ncRNAs present in human saliva as a source of biological information.

## 1. Introduction: Saliva as a Liquid Biopsy

Saliva comes primarily from three major paired salivary glands (parotid, submandibular and sublingual) where specialized cells take up water, salts and macromolecules from the blood that add up to their individual gland secretions. Hence, most compounds found in blood are also present in saliva, which has recently been termed the “mirror of the body” [1,2]. Upon release of glandular secretions into the oral cavity, the fluid is mixed with a variety of exocrine, non-exocrine, cellular, and exogenous components to ultimately form whole saliva (WS). Saliva has a critical role in maintaining the oral health and homeostasis, and the function of the upper gastrointestinal tract. The content of saliva is mostly water but it also contains molecules (posttranslationally modified proteins (e.g., glycoproteins, phosphoproteins), peptides, lipids, minerals, and other small compounds) that lubricate our tongues, thereby facilitating the chewing, speaking and swallowing processes, preventing excessive swings in pH, and beginning the process of digestion [3,4]. Furthermore, saliva protects the oral cavity from foreign invaders, such as bacteria and viruses, by digestion and inhibition of their growth. Unfortunately, the importance of saliva is often appreciated only when it is gone, as commonly happens in patients with oral cancer or undergoing radiation treatments [5].

Saliva is a highly desirable body fluid for biomarker development, as it provides a non-invasive, simple and low-cost method for disease detection and screening [6,7,8]. Many efforts have been made in elucidating the molecular profiles in healthy saliva, both at protein and messenger RNA (mRNA) levels [9]. The overall low concentration of saliva markershindered the development of salivary biomarkers over the last decade. Continuous technological advancements, however, have allowed the performance of high-throughput strategies that overcome this problem. The use of several proteomic techniques, such as 2-D gel electrophoresis, mass spectrometry and Western blot, were used to define the salivary proteome [10,11,12,13,14]. The use of transcriptomic techniques, such as quantitative PCR (qPCR), microarray analysis and deep sequencing analysis have permitted the definition of the salivary transcriptome [15,16,17,18,19,20,21], thereby contributing to the foundation of salivary biomarker development. In addition, the salivary microbiome [22] and metabolome [23,24,25] have also been determined and shown a promising potential as disease-related biomarkers for oral and systemic diseases [22]. The vast amount of “salivaomics” data has led to the development of the the Salivaomics Knowledge Base (SKB) [26], a data management system and Web resource supporting salivary diagnostics research [27,28]. In addition to salivary content, a great deal of effort has been made to standardize procedures for saliva collection, storage and analysis [29,30,31], in addition to methods to increase the stability of the proteins and mRNAs present in saliva [32,33,34,35].

Although saliva fulfills the goal of the holy grail of diagnostics—non-invasiveness—salivary diagnostics is as of yet only recognized for oral diseases; its clinical utility and scientific credibility for systemic diseases is still unsubstantiated. The potential use of saliva has been demonstrated not only for detecting various local diseases, including Sjögren’s syndrome [36,37,38], oral and head and neck cancers [39,40,41,42,43], but also for detecting systemic diseases, such as HIV [44,45,46], hepatitis C virus [47,48,49,50], type 2 diabetes [51], insulin resistance [52], cardiovascular diseases [53,54], lung cancer [55,56,57], resectable pancreatic cancer [58,59], breast cancer [60,61] and ovarian cancer [62].

After a decade of scientific and technological advancements, the incipient maturation of these basic and translational outcomes is leading to the development of clinical tests that benefit patients, based on the use of saliva as a source of biological information. Nonetheless, the majority of all these efforts have been focused on revealing the presence of mRNA and proteins as powerful diagnostic biomarkers, but little is known about the emerging new class of non-coding RNAs (ncRNAs) in saliva body fluid. Here, we review the current status, power, advantages, and future applications of ncRNAs in saliva as a source of biological information, disease status, and biomarker performance.

## 2. Salivary Non-Coding RNAs Associated with Physiological and Pathological States

Around 98% of all transcriptional output in humans is non-coding. RNA-mediated gene regulation is widespread in higher eukaryotes and complex genetic phenomena like RNA interference, co-suppression, transgene silencing, imprinting, methylation, and possibly position-effect variegation and transvection; these are all involved in intersecting pathways based on or connected to RNA signaling [63,64]. Although proteins are the fundamental effectors of cellular function, the basis of eukaryotic complexity and phenotypic variation may lie primarily in a controlled architecture composed of a highly parallel system of trans-acting RNAs: the ncRNAs [63]. The ncRNAs are short RNAs that have been widely described as being stable in many body fluids [65], ostensibly protected from RNA degradation and therefore appearing to be potential biomarkers. The classification of ncRNAs is based on the transcript size: small ncRNAs (<200 bp)—including micro RNAs (miRNAs)—and long ncRNAs (≥200 bp) [63,66,67]. ncRNAs are emerging as new regulators of diverse physiological functions. Importantly, ncRNA deregulation, together with other molecular defects, plays an important role in oncogenesis and tumor progression [68,69,70]. Thus, ncRNAs are very appealing for the development of new target therapies and appear to be important for the discovery of new disease-specific diagnostic makers in body fluids, including saliva.

A complex compositional profile of saliva extracellular RNA (exRNA) has emerged, encompassing mostly mRNAs, but also ncRNAs such as microRNAs (miRNAs), small nucleolar RNAs (snoRNAs), and other non-coding RNAs [21,71,72]. However, despite the large amount of existing data, the entire spectrum of saliva exRNA has not been fully elucidated, thus warranting the need for further comprehensive deciphering analysis. Interestingly, by using high-throughput RNA sequencing (RNA-Seq) and powerful bioinformatic platforms, the landscape of miRNA, piwi-interacting RNA (piRNA) and circular RNA (circRNA) present in human saliva has been described, which represents a new source of interesting biomarkers [73]. In addition, there is also increasing interest in understanding the functional aspects of salivary exRNA in oral and systemic biology [55,59]. Such studies will be facilitated by a detailed delineation of the landscape of salivary exRNA.

### 2.1. Characterization of Salivary Non-Coding RNAs

Compared with other biofluids, saliva can be collected easily and non-invasively. However, low RNA abundance, small sample volumes, highly fragmented mRNA, and high abundance of bacterial content create challenges for downstream RNA sequencing analyses [21]. Thus, ncRNAs rise to the first position of ideal and suitable salivary biomarkers because of its short size, body fluid stability, and their main location inside the exosomes [59,65,72,74].

Among all ncRNAs, small ncRNAs are the most exploited and widely described ncRNAs in saliva, particularly miRNAs, which are small, 19- to 23-nucleotide-long single-stranded RNA molecules. miRNAs play an important role in regulating various biological processes through their interaction with cellular mRNA [75], and have been broadly characterized in saliva body fluid. Weber *et al.* [76], with the goal of assessing the distribution of miRNAs and demonstrating the potential use of miRNAs as biomarkers, examined the presence and distribution of miRNAs in 12 human body fluids, including saliva. They found that miRNAs were present in all the tested body fluids. Moreover, they noticed that several of the highly abundant miRNAs were common among multiple fluid types, and some of the miRNAs were enriched in specific body fluids. Interestingly, saliva, breast milk, and seminal fluid had a higher number of detectable miRNA species, whereas urine, cerebrospinal fluid, and pleural fluid had far fewer. When comparing the miRNA spectrum found in plasma with that from most of the other body fluids, they found important differences, thus indicating an extensive “blood filtering” process that takes place in most body fluids, including saliva. Other discrepancies can be explained by the differential uptake or release of miRNAs from varying circulating cell types that come in contact with the blood, or other processes not yet fully understood.

Different groups have studied the miRNA composition of the WS, including the cell content, cell debris and bacteria present in oral cavity. Patel *et al.* [77] described miR-223, miR-191, miR-16, miR-203, and miR-24 as the five most abundantly expressed miRNAs; they have also been reported in other studies [74,78,79]. Additionally, the improvement of the RNA isolation method increased the recovery of high quality RNA, allowing the detection of many previously undetected miRNAs. Spielmann *et al.* [21] compared the RNA from unstimulated whole saliva (WS) and cell-free saliva (CFS) by using RNA-Seq analysis. They found that more than 90% of the uniquely mapped genes were coding (*i.e.*, mRNAs), and the remaining percentage was comprised of ncRNAs. However, ranking the genes by RPKM (reads per kilobase per million) criteria revealed that 95 out of the top 100 highly expressed genes encode for ncRNAs, most of them classified being as small nucleolar RNAs (snoRNAs). Furthermore, they found measurable differences between CFS and WS; remarkably, a higher fraction of microbial RNA was found in WS compared to CFS, which markedly decreased the sensitivity of human RNA in WS analysis. Therefore, these data suggest that a low-speed centrifugation step reduced the presence of microbial RNA. Adding subsequent steps might therefore remove more of the microbial cells and cell debris. Recently, Bahn *et al.* [73], by using high-throughput RNA-Seq, conducted an in-depth bioinformatic analysis of ncRNAs found in human CFS from healthy individuals, with special focus on miRNAs, piRNAs, and circRNAs. Their data demonstrated robust reproducibility of miRNA and piRNA profiles across individuals. Furthermore, individual variability of these salivary exRNA species was highly similar to those RNAs found in other body fluids or intracellular samples, despite the direct exposure of saliva to environmental impacts. By comparative bioinformatics analysis of more than 90 RNA-Seq datasets of different origins, they demonstrated that piRNAs were surprisingly abundant in CFS compared with other body fluid or intracellular samples; with expression levels in CFS comparable to those found in embryonic stem cells and skin cells. In other words, the most abundant types of small ncRNAs codified for human piRNAs (7.5% of reads), miRNAs (6.0% of reads) and snoRNAs (0.02% of reads). In addition, 58.8% of reads corresponded to microbial RNA sequences, reflecting the enriched presence of microorganisms in saliva [21]. Furthermore, using a customized bioinformatics method, more than 400 circRNAs were found in CFS. These data represent the first global characterization and experimental validation of circRNAs in any type of body fluid [73].

### 2.2. Salivary Exosomes as a Source of Non-Coding RNAs

In an effort to avoid microbial and intracellular RNA contamination, some groups have started to investigate the miRNA content in extracellular vesicles (EVs) isolated from saliva. EVs, commonly known as exosomes, are small membrane vesicles measuring approximately 30 to 100 nm in diameter that carry different molecules—such as proteins, lipids, as well as mRNA and ncRNAs—released from the cell of origin to the microenvironment [80,81,82]. Body fluid-derived EVs are a mixture of vesicles originating from different types of cells: both cells found in the body fluids and/or the cells lining the cavities of extruded body fluids [83,84].

Increasing evidence suggests that RNAs are not passively loaded into EVs, but that certain populations of RNAs become enriched in EVs compared to parental cells, mainly because of a size restriction, but also because of the existence of an active sorting mechanism that occurs at the RNA level [85]. For instance, in cancer development, the release of nucleic acids into the blood seems to be related to the apoptosis and necrosis of cancer cells in the tumor microenvironment; it is also the result of secretion. Circulating RNAs are detectable in the serum and plasma of cancer patients, and are surprisingly stable in spite of the fact that high amounts of RNases circulate in the blood of those patients. This somehow implies that RNA may be protected from degradation by its packaging into EVs [65].

At least for some cell types, miRNAs may be transferred within EVs to neighboring cells, where they alter the gene expression and phenotype of the recipient cells. Moreover, accumulating evidence indicates that the incorporation of miRNAs into EVs allows those miRNAs to circulate in the body fluids while avoiding degradation from RNAse activity. The type and content of salivary EVs suggest that saliva-derived EVs are mainly from epithelial cells and granulocyte origin [86]. Two types of EVs have been identified in saliva: one population that is heterogeneous in their size (30–250 nm), and one population that is homogeneous in their size (20–80 nm) [87].

Currently, EVs isolation is time consuming and therefore it is difficult to consider analyzing salivary EVs for clinical routine. Despite this practical consideration, salivary EVs represent a better method to characterize the exRNA present in saliva. In 2010, Michael *et al.* [74], successfully isolated EVs from human saliva; however, they pointed out that WS viscosity and cellular contamination made it less than ideal for EV characterization. Although several studies have been focused on characterizing salivary EVs at nanostructural, transcriptomic [88,89], and proteomic [90] levels, little is known about ncRNA content in salivary EVs. Gallo *et al.* [91] investigated whether body fluid miRNAs are circulating freely or encapsulated into EVs. They compared the miRNA purified from isolated EVs and the miRNA purified from EV-depleted supernatant, from both serum and saliva body fluids. They found a lower miRNA concentration in salivary EVs than in serum EVs, but still a predominant presence of miRNA in the EVs fraction compared to EV-depleted salivary supernatant. Later on, Ogawa *et al.* [72] investigated the content of small ncRNAs from salivary EVs (two type of salivary EVs were investigated) and from WS, by using RNA-seq technology. They found that many types of small RNA, such as miRNA, piRNA, snoRNAs and other small ncRNAs, are contained in salivary EVs. A total of 143 miRNAs were common in both salivary EVs and WS, while 147 miRNAs were only detected in EVs. Importantly, piRNA and snoRNAs were first described in saliva samples: 129 piRNAs were mostly expressed in salivary EVs, while in WS they only found 90 piRNAs. Furthermore, the number of snoRNAs detected in one EV fraction was less than 50% than in the other EV fraction and WS. Thus, specific ncRNAs appear differentially expressed in WS, depleted or non-depleted EV fraction, and further studies are needed to define the function of small ncRNAs in salivary EVs.

### 2.3. Salivary Non-Coding RNAs as a Source of Biomarkers for Local and Systemic Diseases

The identification of biological markers for specific diseases is a major impetus in current research. An ideal diagnostic biomarker should enable unbiased determination of the disease, especially in patients with early stage disease and without specific symptoms. A good biomarker should fulfill several criteria, such as high specificity, high sensitivity, easy use, standardized protocols, and readability of the results for the clinicians. Theoretically, every disease may be detected and characterized by its unique biomarker. However, a more complete view for a biomarker is a panel of up- and down-regulated molecules that differ in their disease and normal states [92].

Human saliva has been increasingly used for biomarker development to enable non-invasive detection of diseases. The term “salivaomics” was coined to highlight the omics constituents in saliva that can be used for biomarker development and personalized medicine [28]. Salivary exRNA [21] was discovered 10 years ago; since then, the nature, origin, and characterization of salivary exRNA have been actively pursued [17,21,36,40,58]. These studies have demonstrated the potential use of salivary exRNA not only to detect local diseases but also systemic diseases. However, there is a small grouping of studies that reveal ncRNAs as a source of diagnostic biomarkers in saliva either for local or systemic diseases (Table 1).

**Table 1:** Salivary ncRNAs as disease-related molecular markers.

Ref.	Study	Saliva Fraction	Disease	Study Cohort	Technique	Molecular Profile
**Characterization**						
[76]	Weber *et al*., *Clin. Chem.* 2010	CFS	characterization	5 healthy donors	Human miScript Assay panel (Qiagen)—714 miRNA	miR-182*, miR-450b-5p, miR-622, miR-141, miR-26a, miR-145*, miR-135b*, miR-381, miR-96*, miR-1228, miR-431*
[74]	Michael *et al*., *Oral Dis.* 2010	exosomes	characterization	2 healthy donors	miRCURY LNA microRNA Array, v.10.0, (Exiqon, Denmark)	let-7b, let-7c*, miR-128, miR-150*, miR-17, miR-1908, miR-212, miR-27b*, miR-29b, miR-29c, (Top-10)
[77]	Patel *et al*., *Arch Oral Biol.* 2011	WS	characterization	20 healthy donors	TaqMan1 Low Density Array Card (TLDA) Human miRNA Panel v2.0 (Applied Biosystems)	miR-223, miR-191, miR-16, miR-203, and miR-24
[21]	Spielmann *et al*., *Clin. Chem.* 2012	CFS and WS	characterization	8 healthy donors	SOLiDTM Total RNA-Seq Kit and Barcoding Kit (modules 1–16) (Applied Biosystems)	224 snoRNAs
[91]	Gallo *et al*., *PLoS ONE* 2012	exosomes	characterization	Healthy donors (# N/A)	TaqMan MicroRNA Assay, PN 4427975, Applied Biosystems	miR-22, miR202, miR-203, miR-1273d
[72]	Ogawa *et al*., *Biol. Pharm. Bull.* 2013	exosomes and WS	characterization	1 healthy donor (7 saliva collection replicates)	Illumina Genome Analyzer Iix by Hokkaido System Sciences Co., Ltd. (Japan)	miR-378a, miR-143, let-7c, miR-146b, miR-21, let-7f-1, let-7f-2, miR-30a, miR-9-1, miR-9-2, miR-9-3, let-7a-1, let-7a-2, miR-20a, miR-30d, miR-30e; piR-39980, piR-48209, piR-52207, piR-38581, piR-36095, piR-59293, piR-61648, piR-55361; U78, U44, U21, U31, U104, U15A, snR39B
[73]	Bahn *et al.*, *Clin. Chem.* 2014	CFS	characterization	8 healthy donors	Illumina HiSeq 50SE	127–418 miRNAs (Top-2: miR-223-3p and miR-148a-3p)
**Local & Systemic Diseases**						
[78]	Park NJ *et al*., *Clin. Cancer Res.* 2009	CFS and WS	oral squamous cell carcinoma	50 OSCC patients and 50 healthy matched controls	RT-preamp-qPCR	miR-125a and miR-200a
[93]	Wiklund *et al*., *PLoS ONE* 2011	WS	oral squamous cell carcinoma	15 OSCC patients and 7 healthy controls	TaqManH qRT-PCR assays (Applied Biosystems)	miR-375 and miR-200a expression and miR-200c-141 methylation
[94]	Liu CJ *et al*., *Head Neck* 2012	CFS	oral squamous cell carcinoma	45 oral carcinoma, 10 oral verrucous leukoplakia, and 24 healthy controls	TaqMan miRNA assay system (Applied Biosystems, Foster City, CA, USA)	miR-31
[95]	Matse *et al*., *Clin. Cancer Res.* 2013	WS	paratiroid gland tumors	38 malignant tumors and 29 benign parotid gland tumors	TaqMan Human MicroRNA Cards (Applied Biosystems) and RTqPCR	hsa-miR-132, hsa-miR-15b, mmu-miR-140, and hsa-miR-22
[96]	Tang *et al*., *Mol. Med. Rep.* 2013	SP	oral squamous cell carcinoma	4 OSCC saliva samples and 12 healthy controls	RT-qPCR of six lncRNAs found in OSCC tissue	MALALT-1, HOTAIR
[97]	Wang *et al*., *Biosens. Bioelectron.* 2013	CFS	oral squamous cell carcinoma	5 artificial saliva samples (spiked)	Novel home-made electrochemical biosensor magnetic-controllable gold electrode	miR- 200a, miR-142-3p, miR-93 and miR-125a
[98]	Xie Z *et al*., *PLoS ONE* 2013	CFS and WS	esophageal squamous cell carcinoma	NA	Agilent miRNA microarray	miR-144, miR-10b*, miR-21 and miR-451
[99]	Yang *et al*., *BMC Cancer* 2013	SP	oral squamous cell carcinoma	7 non-progressing LGD, 8 progressing LGD into OSCC and 7 healthy controls	The TaqManW low density array (TLDA) qRT-PCR system (Applied Biosystems, Foster City, CA, USA)	miR-10b, miR-660, miR-708, miR-30e, miR-145, miR-99b, miR-181c and miR-197
[100]	Salazar *et al*., *Cell Oncol.* 2014	WS	head and neck cancer	61 HNSCC patients and 61 healthy controls	miScriptTM miRNA microarray, RTqPCR, TCGA	miR-9, miR-134 and miR-191
[101]	Wang *et al*., *Tumor Biol.* 2014	2 saliva data sets, 6 plasma/serum data sets (meta-analysis)	esophageal squamous cell carcinoma	995 ESCC patients and 733 healthy controls	Bioinformatic and Statistics tools	miR-144, miR-10, miR-451
[102]	Momen-Heravi *et al*., *J. Dent. Res.* 2014	CFS	oral squamous cell carcinoma	9 OSCC patients before treatment, 8 patients with OSCC in remission, and 9 healthy controls	NanoString nCounter miRNA expression assay (NanoString Technologies, Seattle, WA, USA)	miRNA-136, miRNA-147, miRNA-1250, miRNA-148a, miRNA- 632, miRNA-646, miRNA668, miRNA- 877, miRNA-503, miRNA-220a, miRNA-323-5p, miRNA-24, miRNA-27b
[103]	Hizir *et al*., *ACS Appl. Mater. Interfaces* 2014	CFS	prostate cancer	NA	nanographene oxide system	miR-21, miR141
[104]	Gao *et al*., *BioMed. Res. Int.* 2014	CFS	pancreatic cancer	30 PC patients and 32 healthy controls	miScript miRNA PCR array human miRNome (384-well plate) (Qiagen)	miR-17, miR-21, miR-181b, miR-196a
**Forensic Science**						
[79]	Hanson *et al*., *Anal. Biochem.* 2009	WS	body fluid identification	Healthy donors (# NA)	RTqPCR	miR-658, miR-205
[105]	Zubakov *et al*., *Int. J. Legal. Med.* 2010	WS	body fluid identification	Healthy donors (# NA)	Microarray LNATM-modified oligo-nucleotides (Exiqon, Vedbæk, Denmark), and RTqPCR	miR-583, miR-518c*, miR-208b
[106]	Courts *et al*., *J. Forensic Sci.* 2011	WS	body fluid identification	Healthy donors (# NA)	Microarray Geniom Biochips (Heidelberg, Germany) and RTqPCR	miR-200c, miR-203, miR-205
[107]	Wang *et al*., *Forensic Sci. Int. Genet.* 2012	WS	body fluid identification	10 healthy donors	RTqPCR	miR-658, miR-205
[108]	Omelia *et al*., *Anal. Biochem.* 2013	WS	body fluid identification	6 healthy donors	RTqPCR	miR-205
[109]	Park JL *et al*., *Electrophoresis* 2014	WS	body fluid identification	60 healthy donors	Affymetrix Gene Chip miRNA 3.0 array and RTqPCR	miR-203, miR-205
[110]	Silva *et al*., *Forensic Sci. Int. Genet.* 2015	–	body fluid identification	–	–	(Review)

WS: whole saliva; CFS: cell free saliva; SP: saliva pellet and NA: not available.

#### 2.3.1. Non-Coding RNAs for Local Diseases

Several studies have been made with the aim of deciphering saliva miRNA profiles for oral cancer early diagnosis. In 2009, Park *et al.* [78], by comparing both CFS and WS from 12 healthy donors and a cohort of 50 cancer patients, found that miR-125a and miR-200a were differentially expressed in patients with oral cancer. In 2011, pursuing the same disease, Wiklund *et al.* [93] described a panel of miRNA and DNA methylation patterns. The panel, consisting of aberrant miR-375 and miR-200a expression and miR-200c-141 methylation, was initially found in oral squamous cell carcinoma (OSCC) tissues and then validated in oral rinse and saliva from OSCC patients and healthy controls, suggesting a potential clinical application for OSCC diagnosis. Later on, in 2012, Liu CJ *et al.* [94] described miR-31 as a clinical biomarker of OSCC in oral lesions, plasma, and saliva. They found miR-31 significantly increased in saliva from patients with oral carcinoma at all clinical stages, including very small tumors. However, no difference on salivary miR-31 expression was found when comparing patients with oral verrucous leukoplakia relative to controls. When comparing miR-31 expression in different body fluids, they found that miR-31 was more abundant in saliva than in plasma, suggesting that salivary miR-31 was a more sensitive biomarker for oral malignancy. Furthermore, they also check the expression of miR-31 after excision of oral carcinoma, and found that salivary miR-31 was remarkably reduced, indicating that most of the up-regulated salivary miR-31 came from tumor tissues. Interestingly, Matse *et al.* [95] have encountered that 95% of the differentially expressed miRNAs were over-expressed in saliva from patients with malignant tumors compared to those of patients with benign parotid gland tumors. A combination of four validated miRNAs was able to discriminate between cancerous and benign saliva, with a sensitivity of 69%, a specificity of 95%, and an area under the curve (AUC) of 0.9.

More recently, Wang *et al.* [97], developed an electrochemical biosensor method for ultra-sensitive and specific detection of oral cancer-related miRNAs at attomolar levels. In order to evaluate the applicability of the novel RNA biosensor, saliva samples were spiked with different concentrations of target miRNAs (miR-200a, miR-142-3p, miR-93 and miR-125a). The results show clear indications that the magnetic-controllable electro-chemical biosensor had a strong resistance to the complex matrix of saliva, and therefore can be used to detect ultra-trace targeted miRNAs in real saliva samples with a recovery of 93%–108%. In 2013, Yang *et al.* [99] reported for the first time the use of miRNA microarray to profile low-grade dysplasia (LGD)-oral premalignant lesions (OPLs) from progressing and non-progressing LGD-OPLs, in order to explore the possible miRNAs that could lead the progression into high-grade dysplasia (HGD) or OSCC. Yang and colleagues identified 25 miRNAs differentially expressed between progressive and non-progressive LGD leukoplakias and, among them, 13 miRNAs were down-regulated and 12 miRNAs were up-regulated in progressive LGD leukoplakias. Moreover, Salazar *et al.* [100] concluded that miR-9, miR-191 and miR-134 can serve as novel non-invasive biomarkers in head and neck squamous cell carcinoma (HNSCC) (*n* = 122), providing a good discriminatory power with AUC values of 0.85 (*p* < 0.0001), 0.74 (*p* < 0.001) and 0.98 (*p* < 0.0001), respectively. In addition, their expression was independently validated using an HNSCC cohort consisting of 334 tumor samples and 39 normal adjacent tissues, curated in the TCGA database, showing a good correlation among tissue and saliva datasets. Finally, Momen-Heravi *et al.* [102] tested more than 700 miRNAs by using NanoString nCounter [111] in saliva samples from OSCC patients. They identified 13 miRNAs that were differentially expressed in OSCC when compared to healthy controls and, among them, 11 miRNAs were down-regulated (miRNA-136, miRNA-147, miRNA-1250, miRNA-148a, miRNA-632, miRNA-646, miRNA-668, miRNA-877, miRNA-503, miRNA-220a, miRNA-323-5p), and two miRNAs were over-expressed (miRNA-24, miRNA-27b).

Recently, a meta-analysis study was published addressing esophageal squamous cell carcinoma (ESCC) [101]. The study included six datasets from plasma/serum circulating miRNAs and two datasets from a saliva miRNA profile for ESCC; all datasets belong to previous studies published on the field [98,112]. Moreover, 17 studies, including 995 ESCC patients and 733 healthy controls, were included in the analysis. The meta-analysis showed a pooled AUC of 0.91 (95% CI 0.88–0.93). A sub-meta-analysis indicated that a blood-based miRNA assay displays better diagnostic accuracy than a saliva-based miRNA assay. Nevertheless, the individual study reported by Xie *et al.* [98], where the data for meta-analysis is derived from, showed that miR-10b*, miR-144, and miR-451 in WS, and miR-10b*, miR-144, miR-21, and miR-451 in CFS were significantly up-regulated in patients with ESCC, with sensitivities of 89.7%, 92.3%, 84.6%, 79.5%, 43.6%, 89.7%, and 51.3%, and specificities of 57.9%, 47.4%, 57.9%, 57.9%, 89.5%, 47.4%, and 84.2%, respectively, therefore demonstrating that saliva miRNAs possess discriminatory power for detection of ESCC.

Most of the studies cited above described small ncRNAs, specifically miRNAs, the most common and exploited biomarkers found in saliva. However, there is only one study that describes long ncRNAs in saliva for oral cancer detection [96]. In this study, some of the detected long ncRNAs were aberrantly expressed in OSCC and metastatic tissues. Moreover, when checking the expression of these long ncRNAs in WS some of them appeared to be potential salivary biomarker candidates. Among several long ncRNAs investigated, MALAT-1 was present in all the patients investigated (*n* = 9), but HOTAIR was only detected in five out of nine patients with higher expression than in patients with lymph node metastasis. Taken together, this data indicates that WS contains a detectable amount of certain long ncRNAs that may be potential markers for OSCC diagnosis.

#### 2.3.2. Non-Coding RNAs for Systemic Diseases

To our knowledge, there are only two studies that focused on salivary and circulating ncRNAs. Firstly, Hizir M.S. and colleagues [103] evaluated the expression of miR-21 and miR-141, two miRNAs over-expressed in early and advanced prostate cancer patients, respectively. They have demonstrated simultaneous detection of endogenous and exogenous miR-21 and miR-141 from human body fluids including blood, urine, and saliva, by using a specially designed biosensing nanographene oxide system, which uses two different wavelengths to detect miR-21 and miR-141 simultaneously (520 and 670 nm, respectively). Their approach is faster and easier than multiple and simultaneous qPCR detection, and can be performed with a portable spectrofluorometer; however, the sensitivity needs to be improved for clinical application. Secondly, Gao *et al.* [104] described a three-miRNA panel (miR-17, miR-21, and miR-181b) in CFS that improves the current techniques of traditional Chinese medicine (TCM) to diagnose pancreatic cancer. A cohort of 62 patients was subsequently confirmed by a larger cohort based on several online databases. Thus, these studies represent the beginning of the salivary small ncRNAs’ utility to diagnose systemic diseases through a non-invasive method. However, no study has been published in the field of salivary long ncRNAs and non-oral diseases. Since there is much evidence that long ncRNAs can serve as circulating diagnostic biomarkers for several diseases, like B-cell neoplasms and prostate cancer [113,114,115], and also that those long ncRNAs have sufficient power to discriminate between cancer and healthy status [96,116], we truly believe that it is just a matter of time before long (and short) ncRNAs will appear as the new spectrum of diagnostic biomarkers in saliva, specific for local and systemic diseases, reaching the same position as transcriptomics and proteomics, which are currently leading in salivary diagnostics.

### 2.4. Forensic Science and Body Fluid Identification

Forensic scientists are interested in detecting the presence of tissue-specific mRNAs in dried body fluid stains, including saliva, as a means of positively identifying the tissue source of origin of a physiologic stain recovered from a crime scene [79,117,118,119]. They have not expressly determined whether the varieties of mRNA species that are routinely detected in saliva originate from the cellular or non-cellular fractions of saliva because, in a forensic context, the cellular *vs.* non-cellular source is immaterial. Nevertheless, the presence of mRNA species in WS is irrefutable and they routinely isolate mRNA of sufficient quality and quantity [117] for routine RT-PCR and RT-qPCR analyses. The persistence and stability was such that they could detect and analyze mRNA from small quantities of dried saliva stains collected weeks or even months after the initial deposition. They have identified a number of candidate tissue-specific genes (statherin, histatin 3, PRM1, PRM2), which may be useful for the positive identification of saliva. mRNAs from these genes were detectable in saliva stains but not in blood or semen stains [117,118]. Collectively these findings constitute the basis of a prototype RNA-based assay system that may eventually supplant conventional methods for body fluid identification.

In addition to the efforts devoted to look for forensic salivary mRNA, several studies have been recently focused on ncRNAs following the same purpose. The size of the amplification products used in the mRNA assays (>200–300 nt) might not be ideal to use with degraded or compromised samples frequently encountered in forensic casework. Therefore, there has been an explosion of interest in a class of small ncRNAs: miRNA. Hanson *et al.* [79] provided the first comprehensive evaluation of miRNA expression in dried, forensically relevant biological fluids—blood, semen, saliva, vaginal secretions, and menstrual blood—in an attempt to identify putative body fluid-specific miRNAs. They have identified a panel of nine miRNAs—miR-451, miR-16, miR-135b, miR-10b, miR-124a, miR-372, and miR-412, miR-658, miR-205 (being the last two specific of saliva)—that are differentially expressed to such a degree as to permit the identification of the body fluid origin of forensic biological stains using as little as 50 pg of total RNA. Courts *et al.* [106] and Park *et al.* [109], after microarray analysis, also validate by RT-qPCR one of the selected miRNAs that Hanson and colleagues found specific to saliva body fluid identification. Conversely, Zubakov *et al.* [105] have selected a total of 14 most promising biomarkers derived from a microarray data analysis for validation in TaqMan-based RT-qPCR assays, but they did not find a correlation between their microarray data and RT-qPCR expression analyses for saliva, vaginal secretions, and menstrual blood. The failure to include all forensically important body fluids in genome-wide miRNA profiling may be a cause for discrepancy among these studies [109]. Apart from that, Wang *et al.* [107] created an efficiency-calibrated model that incorporated the impact of the quantification cycle (Cq) values and PCR efficiencies of target and reference genes to calculate the relative expression ratio of miRNAs in forensically relevant body fluids, suggesting that miR-658 and miR-205 were non-specific for saliva among the other forensically tested body fluids. Furthermore, Omelia *et al.* [108] confirmed that it is possible to detect miR-205 in saliva samples that were previously extracted using standard DNA extraction protocols, suggesting the usefulness of these previously tested forensic samples. Considering all of the above, Silva *et al.* [110], very recently reviewed the multiple factors that have the potential likelihood of discrediting miRNA profiling, and highlight the ultimate question whether miRNA profiling can be used or not as the forensic biomarkers for body fluid identification.

## 3. Salivary Non-Coding RNAs as a Diagnostic Test Tool

The utility of saliva as a diagnostic tool, which provides a non-invasive, easy, and low-cost method for disease detection and screening, has been reviewed elsewhere [120]. Saliva collection is more practical and comfortable compared with other invasive methods, and that is one of the main reasons that make saliva a desirable body fluid for clinical applications, with particular interest in large population screening, children, geriatric patients, and in cases where repeated samplings are needed. The growth of the knowledge in constituents within saliva has helped the rapid and accurate detection and quantification of its molecules, allowing researchers to discover, develop, and validate biomarkers for detection of several diseases. Thus, clinical salivaomics has much more promise in medicine with cutting-edge omic technologies combined with advanced bioinformatics. However, without proper study design and implementation of robust analytical techniques, the efforts and expectations may very easily be hampered.

Saliva is a proximal body fluid in the oral cavity and therefore is intuitively sound for detection of oral diseases. However, this increasingly used body fluid has been recently termed “liquid biopsy”, useful to detect non-oral diseases [51,55,56,57,58,59,60,61,62]. Some studies hypothesize that molecules can travel from the primary tumor to the blood and appear altered in cancer compared to control saliva. Gao *et al.* [55] used induced tumor bearing mice models of melanoma and lung cancer and defined a salivary transcriptome profile associated to each tumor-type. Lau *et al.* [59], by using a pancreatic tumor-bearing mice model, revealed the basic mechanisms underlying the rationale of salivary biomarkers through the hypothesis that exosome-like vesicles carry, drive, and deliver tumor markers into the saliva. The lack of clear mechanisms showing how salivary biomarkers can reflect disease states elsewhere in the body has compromised the scientific acceptance of this emerging field [28]. Although several hypotheses have been proposed, the mechanistic underpinning awaits validation [28]. The clinical and scientific credentialing of saliva for systemic disease detection will present a groundbreaking technology, impactful and sustainable, that will transform molecular diagnostics globally.

As previously mentioned, whilst several efforts have been made to characterize small ncRNAs in saliva, there is no such information for long ncRNAs. There is a necessity to improve standardized protocols for long ncRNA isolation, data analysis, and bioinformatics pipelines that yield better quality of starting materials and significant data, which will therefore allow characterization and good quality outcomes. Thus, the emerging interest in the field and the recent publications revealing new ncRNAs in saliva, including miRNAs, piRNAs, and circRNAs, may be, in the near future, the basis for future biomarker discoveries.

## 4. Conclusions and Future Perspectives

**Figure 1:**
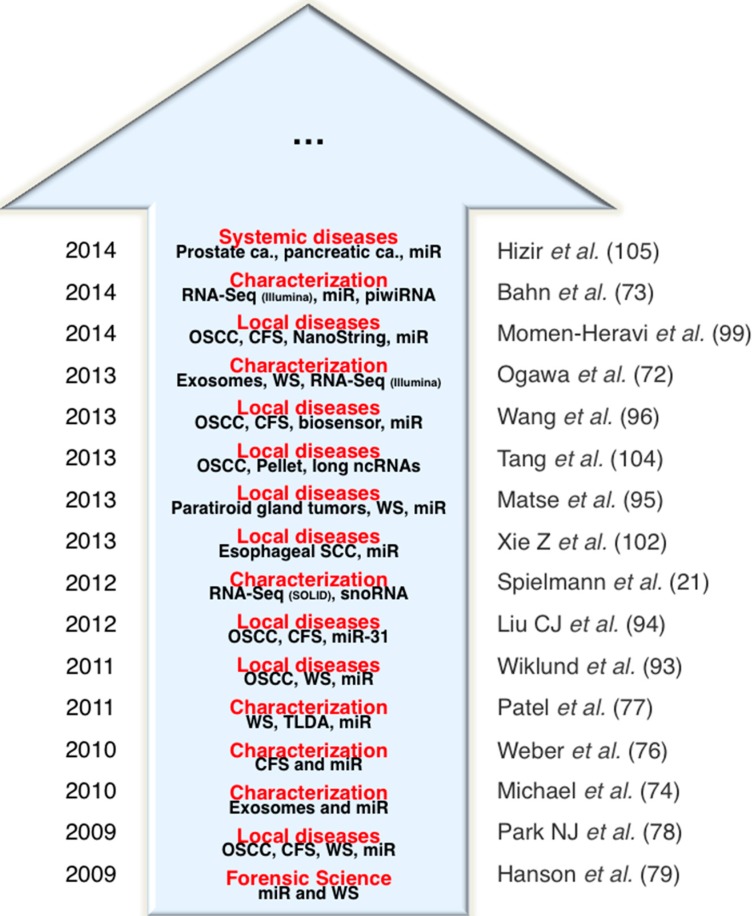
The rise of non-coding RNA in saliva.

ncRNAs profiles in human diseases, especially in cancer, have highlighted the potential value of this class of RNAs as disease-related biomarkers for patient diagnosis. The rapidly expanding and continuous cataloging of salivary ncRNAs holds promises that, in the near future (Figure 1), ncRNAs will become even more important in disease–patient management. An analogy can be made with the impact of salivary mRNA profiling in many types of disease, which has provided different experimental lines of evidence that deregulation of mRNAs not only results as a consequence of cancer progression, but also directly affects gene networks that promote tumor initiation and progression in a cause–effect manner.

As the catalog of salivary ncRNAs grows, it will become important to elucidate the genetic networks and pathways regulated by the abnormally expressing ncRNAs in saliva from cancer patients as a means to understand the role and biomarker performance of these ncRNAs in the induction of malignant transformation, as well as their ability to create significant profiles for salivary diagnostics. In summary, ncRNAs profiles could have an impactful applicability to clinical practice by meeting the demand for an inexpensive, non-invasive, and accessible diagnostic tool.
